# Exploring the Genetic Diversity, Virulence and Antimicrobial Resistance of Diarrhoeagenic *Escherichia coli* From Southern Africa Using Whole‐Genome Data

**DOI:** 10.1002/puh2.70098

**Published:** 2025-08-12

**Authors:** Josphat Gichure, Tina Hald, Elna Buys

**Affiliations:** ^1^ Department of Consumer and Food Sciences University of Pretoria Pretoria South Africa; ^2^ National Food Institute Technical University of Denmark Lyngby Denmark

**Keywords:** antibiotic resistance, diarrhoeagenic *Escherichia coli*, Mozambique, virulence, whole‐genome sequencing

## Abstract

**Introduction**: Previous studies, including our research, provide critical insights on the contamination of food, water and environment in the Southern African Development Community (SADC) with diarrhoeagenic *Escherichia coli* (DEC). This study used whole‐genome sequencing to investigate the genetic diversity, virulence‐associated factors and antimicrobial resistance (AMR) patterns of DEC isolated from children under 5 years old and food sources in Maputo and compared these findings with publicly available DEC genome assemblies from the Southern Africa region.

**Methods**: Whole‐genome sequence data from 11 DEC isolates from food, children under 5 and water sources in Maputo, Mozambique, were analysed alongside 125 publicly available DEC genomic assemblies from the SADC region. The latter were retrieved from the EnteroBase database (http://enterobase.warwick.ac.uk) and included isolates previously collected from food, animals and environmental sources. Genomic analyses were performed using the online pipelines provided by the Centre for Genomic Epidemiology (CGE), Denmark. Unsupervised hierarchical clustering was applied to visualize patterns in genetic diversity, AMR, virulence‐associated genes and plasmid content using the R software.

**Results**: Clustering based on single nucleotide polymorphism (SNP) and core genome multilocus sequence typing (cgMLST) alleles revealed associations based on geographic locations, sample niche, pathovar and O:H antigen, pointing to evolutionary relatedness between the clades with principal coordinate analysis uncovering this accounted for 27.55% of the genetic diversity. Virulence‐associated genes encoding for attaching and effacing (eae) (63.97%), heat‐labile toxin (LT) (25.00%) and Shiga toxin 1 (Stx1) (15.44%) were most abundant, with an inverse association between genes encoding for the presence of LT and eae. Resistance to folate pathway antagonists (sulfamethoxazole—55.9%), β‐lactamases (amoxicillin, ampicillin and piperacillin—all 54.4%) and aminoglycoside (streptomycin—55.1%) was most abundant.

**Conclusions**: The study revealed region‐specific lineages, evidence of horizontal gene transfer and the clustering patterns suggest both localized and cross‐border transmission. The study provides insightful evidence on DEC transmission patterns associated with antimicrobial and disinfectant resistance and associated virulence factors.

## Graphical abstract

Whole genome insights into diarrhoeagenic *Escherichia coli* (DEC) strains from Southern Africa

→ Study focus: Comparative genomics of DEC strains isolated from food, clinical and environmental sources in Southern Africa.

**Key Findings**:

1. High genetic diversity

→ Region‐specific distribution system observed from association trends between strains from similar geographic locations, sample niche, pathovar and O:H antigen, pointing to evolutionary relatedness between the clades.

2. Virulence gene profiles

3. Antimicrobial resistance (AMR)

→ Resistance to folate pathway antagonists (sulfamethoxazole—55.9%), β‐lactamases (amoxicillin, ampicillin and piperacillin—all 54.4%) and aminoglycoside (streptomycin—55.1%) were most abundant.

→ Antimicrobial resistance patterns among the DEC strains suggest horizontal gene transfer within similar geographical locations in Southern Africa.

**Regional relevance**:

→ Highlights the public health risk posed by DEC in food systems and human populations across the Southern African Development Community (SADC) region.

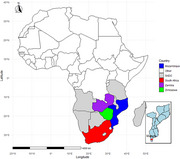

## 1 Introduction

Diarrhoeagenic *Escherichia coli* (DEC) remains a significant cause of acute gastroenteritis in the Southern African Development Community (SADC) [1, 2]. Previous studies, including our research (the Foodborne Disease Epidemiology, Surveillance and Control in African LMIC‐FOCAL project in Mozambique, Tanzania, Ethiopia and Nigeria), have provided critical insights into the contamination of food and water consumed by children, uncovering the dynamics that influence the transmission of DEC [3, 4]. Despite these advances, questions regarding genetic diversity, antimicrobial resistance (AMR) and virulence factors remain unresolved to expound our understanding of how genetic diversity correlates with source attribution and transmission in Mozambique. This calls for using comparative data from SADC to understand local bacterial evolution, resistance mechanisms and virulence factors. Growing concerns about multidrug resistance (MDR) and extended‐spectrum β‐lactamases (ESBLs) producing DEC pose a public health risk [5, 6]. Limited local genomic data hinder the development of tailored diagnostic tools and practical treatment guidelines in SADC.

Whole‐genome sequencing (WGS) has improved surveillance of pathogens in point‐source outbreak investigation, tracking the spread of virulence and antimicrobial‐associated resistance genes to the food and environment. Surveillance studies often lack predefined labels or groupings, making detection of emerging trends or novel variants difficult. Unsupervised hierarchical clustering and *k*‐means of WGS data have uncovered intrinsic genetic patterns, grouping isolates based on genetic similarity without prior knowledge. This enables the detection of natural subpopulations that may correspond to distinct lineages, outbreaks or resistance patterns [7, 8].

Despite the significant burden of diarrhoeal disease in Southern Africa, there is limited genomic data on DEC circulating in the region, hindering effective public health interventions. This study addressed a critical gap by analysing the genetic diversity, virulence‐associated determinants and AMR profiles of DEC isolates using whole‐genome sequence data from children under 5 years old who had diarrhoea and food sources in Maputo, Maputo, and compared these with publicly available DEC genomic assemblies from the SADC region previously collected from food, animals and environment. Such insights are essential for understanding transmission dynamics, identifying locally circulating lineages and detecting emerging resistance threats. This regional context ensures that data from Mozambique—particularly on unique DEC genetic markers and resistance genes found in Maputo—provides a foundational resource to support antimicrobial stewardship, outbreak preparedness and regional collaboration in managing DEC‐related public health threats across the SADC region.

## 2 Methodology

### 2.1 WGS Data Sources

**FIGURE 1:**
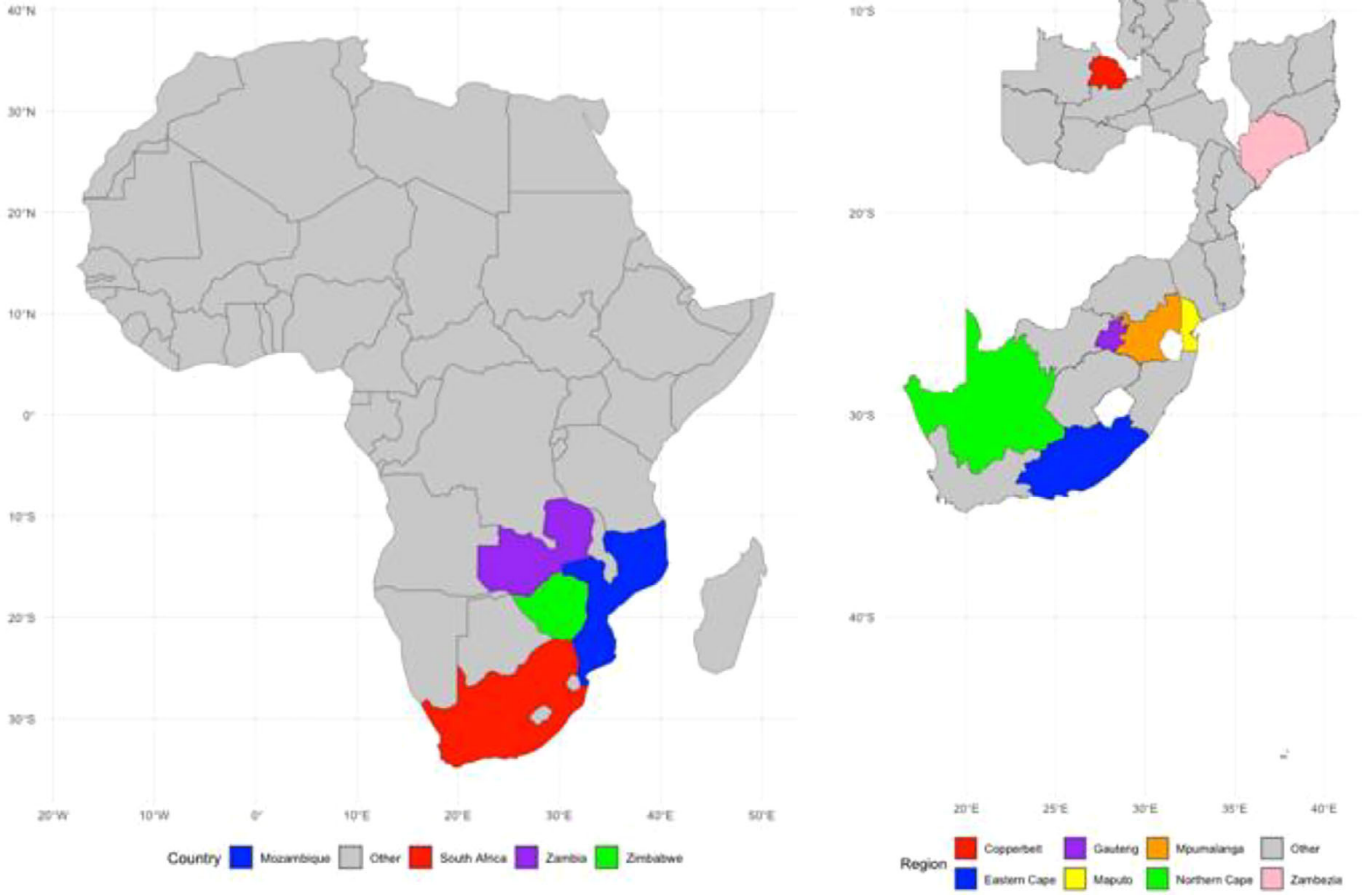
Part (A) highlights the map of Africa with countries that had DEC assemblies publicly available in the EnteroBase database. Part (B) highlights the regions within the countries.

Eleven DEC isolates, human (7) and food (4), obtained from Maputo, Mozambique, were collected and confirmed as described in our previous research (Table S1) [3, 4]. WGS was done with the MGI DNBSEQ‐G400 sequencing instrument using the MGIEasy Universal DNA Library Prep Kit (https://en.mgi‐tech.com/products/reagents_info/id/8) protocol at the Agricultural Research Council (Onderstepoort, Pretoria, South Africa). The non‐interleaved paired‐end FASTQ sequence files were uploaded to Kbase (https://www.kbase.us/), an online web‐based platform [9]. FastQC (v0.12.1) was used to assess sequence file quality, whereas trimming and removal of the adapters from the reads were done using Trimmomatic (v0.36). De novo assembly was used to align the short reads into contigs using SPAdes (v3.15.3) [10]. The non‐interleaved paired‐end FASTQ sequence files were also uploaded to EnteroBase v1.2.0 (http://enterobase.warwick.ac.uk) database web‐based platform [11]. The presence of virulence‐associated factors encoding for Shiga toxin 1 (Stx1), Shiga toxin 2 (Stx2), invasion plasmid antigen H (*ipaH*), invasion plasmid (pInv), heat‐stable enterotoxin (ST), heat‐labile enterotoxin (LT) or intimin (eae) was used to confer the pathovar. One hundred and twenty‐five DEC assemblies from the Southern Africa region that were publicly available in the EnteroBase database were also obtained. The metadata comprising the uberstrain number, assembly barcode, source details, collection year, rST, MLST, core genome multilocus sequence typing (cgMLST), wgMLST, pathovar, Clermont Typing and O:H antigen of the 136 DEC isolates analysed in this study has been attached in the Supporting Information section. The assemblies, single nucleotide polymorphism (SNP) matrix and alleles obtained from the EnteroBase database were downloaded for downstream processing. The map of the Southern Africa region from which the DEC assemblies were obtained is presented in Figure 1A, whereas Figure 1B highlights the regions within these countries. The unknown regions have not been included, whereas Coutada 11 is in Zambezia province in Mozambique.

### 2.2 Analysis of Genome

The assemblies of the DEC isolates were analysed using a web‐based pipelines at the Centre for Genomic Epidemiology (CGE), DTU, Denmark (https://www.genomicepidemiology.org/services/). Core genome alleles were obtained using cgMLSTFinder 1.2 [11, 12]. ResFinder 4.5.0 was used to identify acquired antimicrobial and disinfectant resistance genes and chromosomal point mutations based on the following genes: *gyrA, gyrB, parC, parE, pmrA, pmrB, folP*, 23S, 16S‐*rrsB*, 16S‐*rrsC*, 16S‐*rrsH*, *ampC*‐promoter‐size‐53 bp, *rpoB* [13, 14]. PlasmidFinder 2.1 was used to identify plasmids that confer antibiotic and disinfectant resistance [14, 15].

*E. coli* str. K‐12 substr. MG1655 obtained from the NCBI website was used as the reference genome to confer phylogeny as recommended [16]. SNP analysis and identification of the phylotypes, MLST, cgMLST, rMLST and serotypes were performed using EnteroBase with all the parameters set at default [11]. Hamming distance was used to perform a pairwise distance matrix between the cgMLST alleles with the unweighted pair group method with arithmetic mean (UPGMA) algorithm used to perform hierarchical clustering [17] in R with 100 bootstraps to enhance the reliability of the tree nodes. Clustering the SNP was performed using the Neighbour‐Joining algorithm [17]. For MLST, alleles of seven housekeeping genes, *adk*, *fumC, gyrB*, *icd*, *mdh*, *purA* and *recA*, were used. The serotype of the *E. coli* genome was performed using EnteroBase, based on the O‐antigen (lipopolysaccharide) and H‐antigen (flagellar) genes [18].

The alleles and annotations of the core genomes were analysed using *R* statistical environment (V 4.2.2), ape (v 5.6‐2), phangorn (v 2.11.1), pvclust (v 2.2‐0) and compGenomeRData (v 0.1.0), whereas visualization was done using dendextend (v 1.17.1) packages.

## 3 Results and Discussion

### 3.1 Genetic Diversity Clustering Patterns

#### 3.1.1 Phylogenetic Analysis on SNP and cgMLST

**FIGURE 2:**
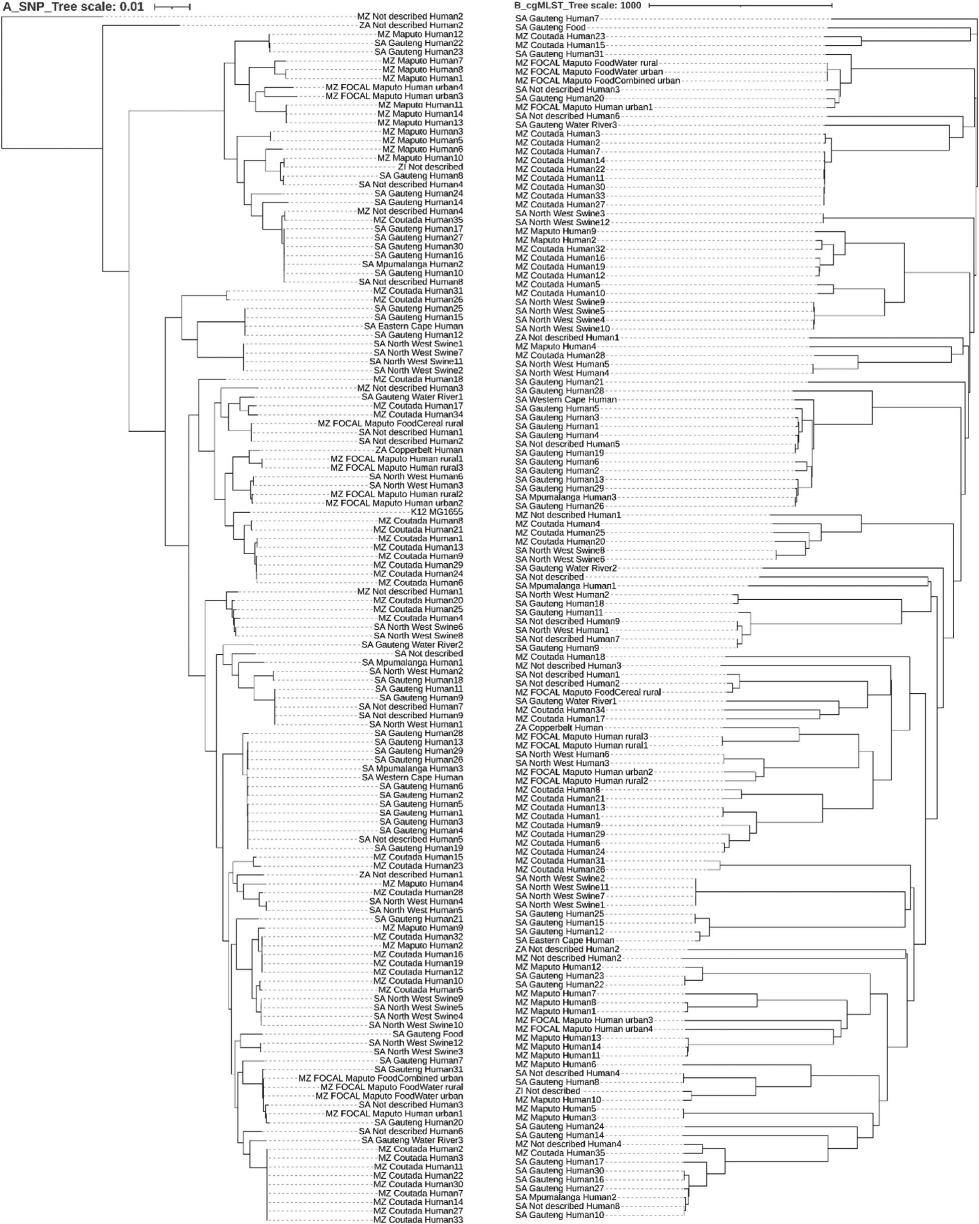
Maximum likelihood phylogenetic trees using SNP (A) and using cgMLST (B) of DEC from Southern Africa. The names have three parts: ZA = Zambia, MZ = Mozambique, SA = South Africa and MZ_FOCAL = the Mozambique FOCAL project. The second part of the names = the provinces (regions) within these countries. The third part = the source of the samples. cgMLST, core genome multilocus sequence typing; DEC, diarrhoeagenic *Escherichia coli*; SNP, single nucleotide polymorphism.

A side‐by‐side visual comparison of dendrograms generated using SNP and cgMLST amongst the 136 DEC isolates from the Southern Africa region has been presented in Figure 2A using SNP matrix and Figure 2B using cgMLST. For SNP, 341,044 variant sites and 3068 accessory genomes were identified, with the minimum percentage sites maintained at 95%, whereas 2513 alleles of core genomes were identified for cgMLST. There was evidence of isolates from similar geographical locations clustering revealing localized transmission patterns evolution. The side‐by‐side comparison of SNP and cgMLST dendrograms clustered the isolates with a fairly similar trend observed.

A complete description and STs of the DEC isolates with the metadata have been presented in the Supporting Information section (Supplementart Data 2, Supplementart Data 1, Figure S1). Sixty‐three unique STs were identified from which ST 21 (*n* = 13, 9.56%), ST 443 (*n* = 9, 6.62%), ST 583 (*n* = 8, 5.88%) and ST 40 (*n* = 7, 5.15%) were most abundant (Figure 4). The isolates were further categorized on the basis of the ST complex (Cplx), where 97 were identified, whereas 39 were unknown. ST 29 Cplx (*n* = 14, 14.43%), ST 278 Cplx (*n* = 12, 12.37%), ST 205 Cplx (*n* = 10, 10.31%), ST 122 Cplx (*n* = 9, 9.28%) and ST 10 Cplx (*n* = 8, 8.25%) are the most abundant complexes. On the basis of rMLST, 77 unique profiles with rST 2264 (*n* = 11), rST 2199 (*n* = 9), rST 1523 (*n* = 8) and rST 14908 (*n* = 4) are the most abundant. On the basis of cgMLST, 121 unique cgSTs were reported, with 15 cgST duplicates and none appearing more than twice. All the 136 wgMLST profiles were unique.

The phylogenetic relationships based on SNPs amongst the 136 DEC isolates based on hierarchical clustering using the UPGMA algorithm, where each point represents an isolate and colours indicate cluster membership, have been presented in Figure S2. The optimal number of *k*‐means nine clusters determined using the Elbow method has been presented in Figure S3. The number of DEC isolates in each cluster has been presented in Figure S2. The hierarchical relationships of 136 DEC isolates from the Southern Africa region based on their cgMLST alleles profiles have been presented in Figure S4. The *p* values of each branch within the clusters have been presented in Figure S5. The bootstrap results on the consistency of cgMLST cluster assignment have been presented in Figure S6.

From both algorithms, the clusters were predominantly associated with geographic region, MLST, pathovar and O–H antigen, pointing to potential epidemiological linkage. In addition, genetic dissimilarities were observed among samples from different source niches within similar geographical locations. To validate the robustness of *k*‐means clustering, the study performed hierarchical clustering and observed consistent subgroup patterns, reinforcing the reliability of the *k*‐means clusters. *K*‐means clustering assumes spherical clusters; therefore, there is a need to capture the underlying genetic relationships to generalize groupings fully [19]. These findings highlight the need to integrate genetic diversity among DEC isolates with epidemiological surveillance in DEC outbreak investigations.

**FIGURE 3:**
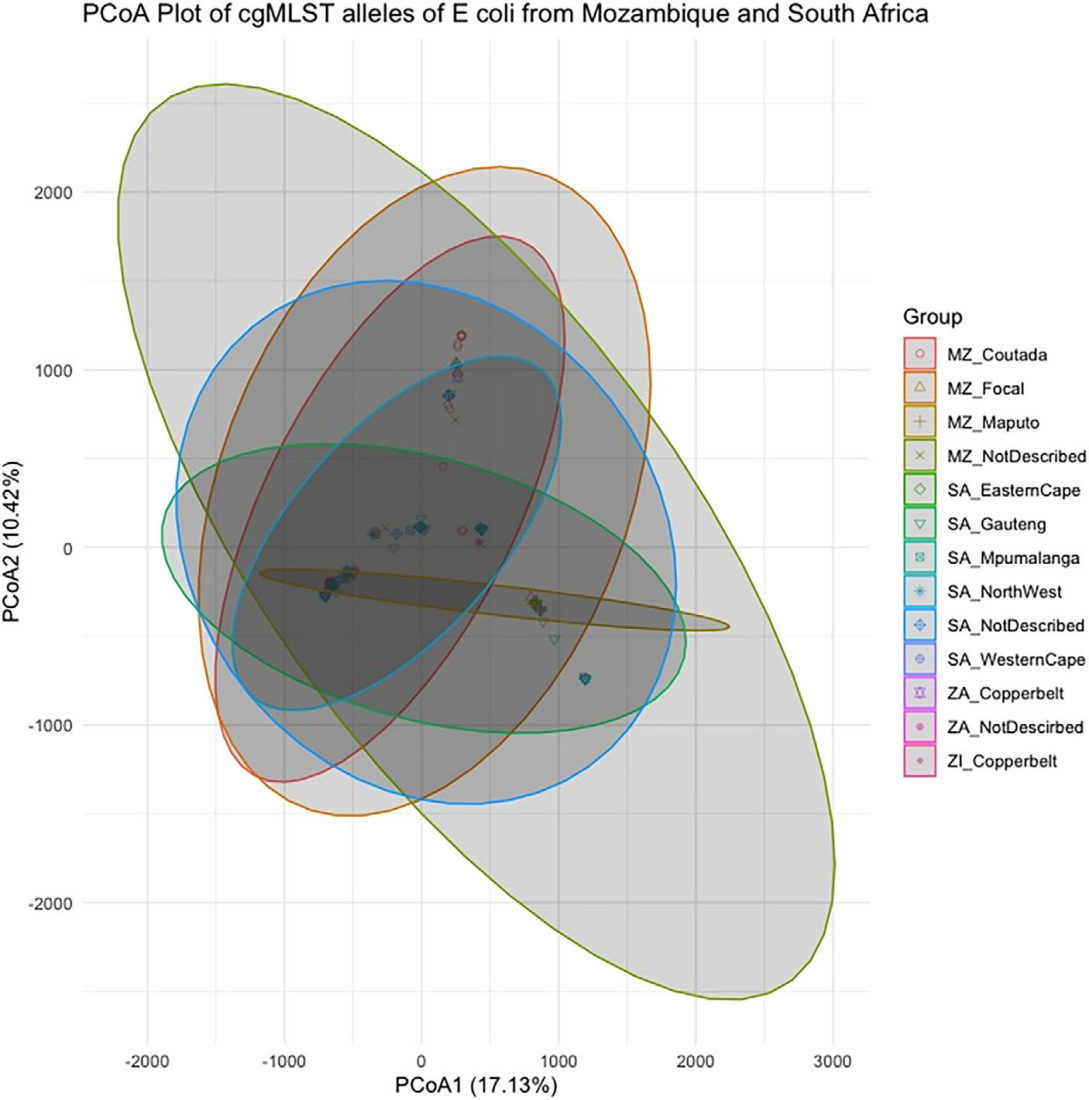
Principal coordinate analysis of cgMLST alleles of 136 DEC strains from the Southern Africa region based on Hamming distance. The names have two parts: ZA = Zambia, MZ = Mozambique, SA = South Africa and MZ_FOCAL = the Mozambique FOCAL project. The second part of the names = the provinces (regions) within these countries. PCoA, principal coordinate analysis; cgMLST, multilocus sequence typing.

The principal coordinate analysis (PCoA) of the DEC isolates has been presented in Figure 3. The cgMLST analysis shows that 17.13% and 10.42% of the total genetic variability could be attributed to PCoA1 and PCoA2, respectively. The ellipse further revealed that the genetic diversity of isolates from similar geographical locations tends to cluster together.

From these clustering algorithms, geographical differentiation played a primary role in the clustering of local clones. As reported in other studies, phylogenetic clustering was not affected by epidemiological factors within the region [19].

#### 3.1.2 Association Between Diversity and Metadata

**FIGURE 4:**
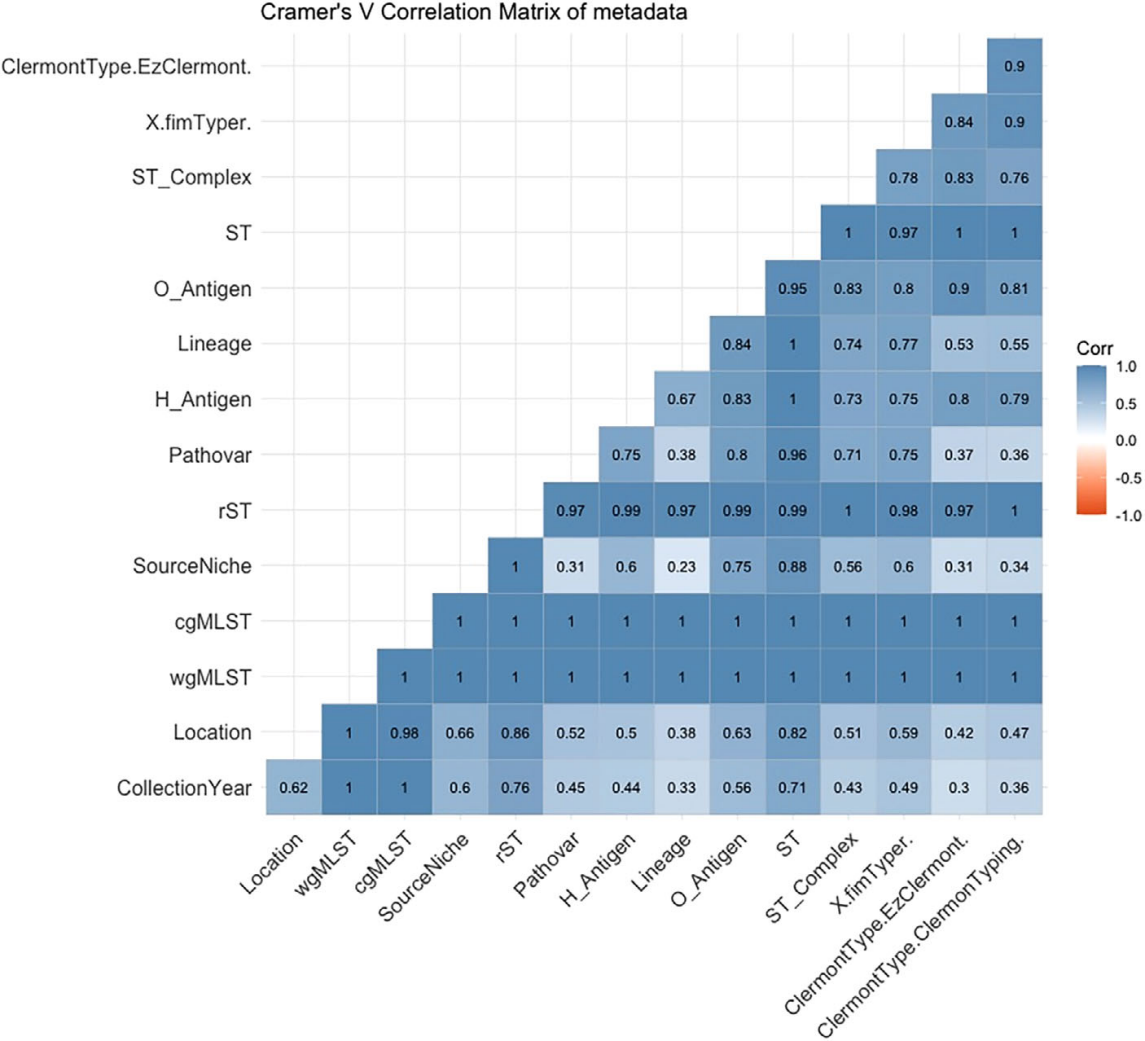
The correlation matrix of metadata of WGS data of DEC isolates alleles from the Southern Africa region. cgMLST, core genome multilocus sequence typing.

The strength and significance of associations of variables within the metadata have been presented in Figure 4. The moderate association between the pathovar and the collection year, geographical location and source niche indicates no direct linkage with pathovars. The presence of local clades was observed as the location had an increasing association as the number of alleles increased, with wgMLST having a solid association, whereas MLST had the least, which indicates that genetic diversity was strongly associated with geographic location.

### 3.2 Virulence and Resistance Associated Genes Clustering Patterns of the DEC Isolates

#### 3.2.1 Virulence Factors Profiling

**FIGURE 5:**
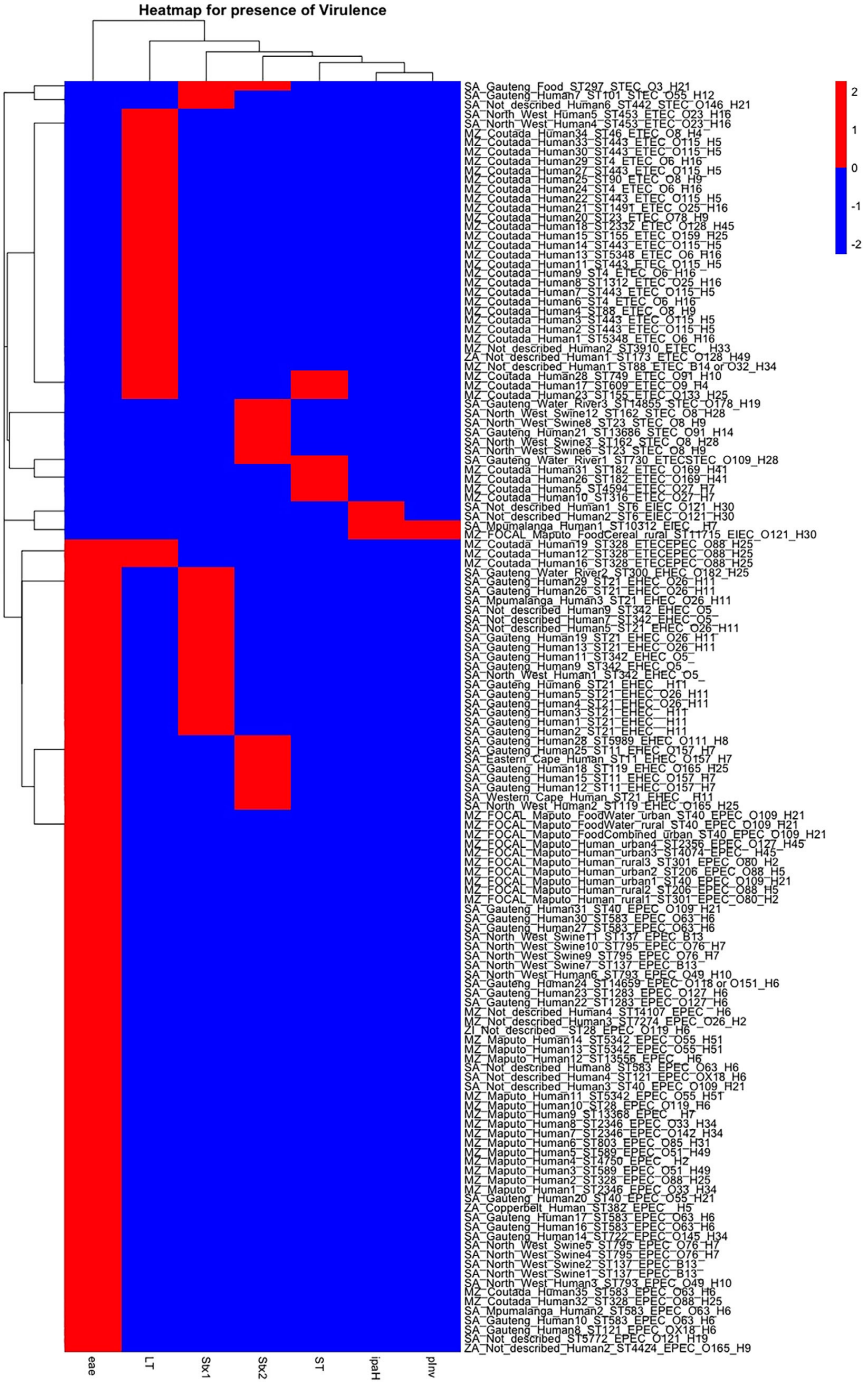
Heatmap on the presence of virulence factors for DEC isolates in Southern Africa. The names have five parts: ZA = Zambia, MZ = Mozambique, SA = South Africa and MZ_FOCAL = the Mozambique FOCAL project. The second part of the names = the provinces (regions) within these countries. The third part = the source of the samples. The fourth = the ST number. The fifth part = the pathotype. The sixth part = the O:H antigen.

A heatmap on the presence of virulence‐associated genes for DEC isolates from the Southern Africa region is presented in Figure 5. Of the 136 DEC isolates, 36 had two virulence‐associated genes, whereas the rest had just one. The most abundant virulence‐associated genes encode for eae (63.97%), LT (25.00%), Stx1 (15.44%), Stx2 (11.76%), ST (5.88%), *ipaH* (2.94%) and pInv (1.47%). Clustering revealed that geographical location was associated with virulence. Fifty‐eight of the DEC isolates were EPEC, 35 were ETEC, 26 were EHEC, 9 were STEC, 4 were EIEC, and 3 were ETEC/EPEC, whereas 1 was ETEC/STEC.

**FIGURE 6:**
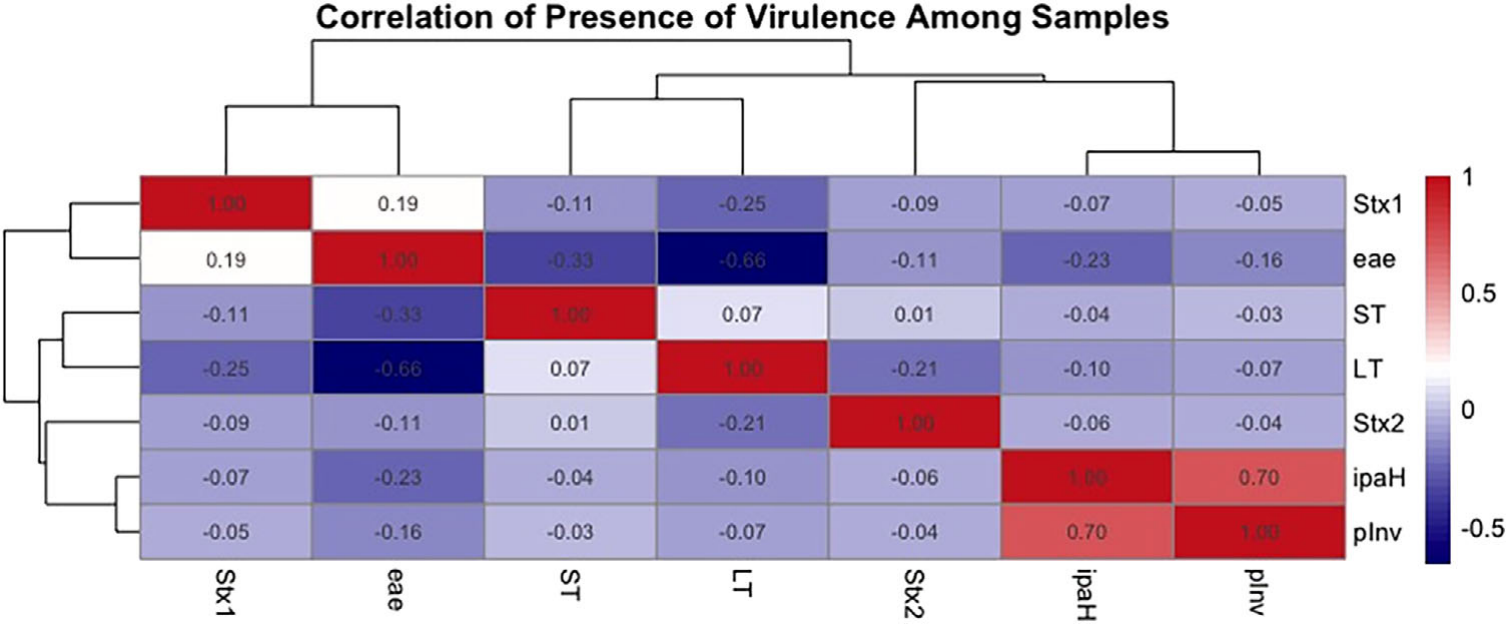
Correlation values on the presence of virulence factors among DEC isolates from the Southern Africa region.

The correlation matrix showing the presence of virulence‐associated genes among the DEC isolates has been presented in Figure 6. A negative correlation was observed between the presence of genes encoding for eae and Stx1 and other virulence‐associated determinants, whereas presence of genes encoding *ipaH* and pInv had a positive relationship. These patterns suggest potential horizontal transfer of virulence genes within DEC clades from similar geographic regions, possibly driven by local selective pressures. Although gene co‐occurrence has been documented in AMR studies, comparable data for DEC remain limited. These findings may support the hypothesis of co‐virulence, as previously proposed, where the combined presence of multiple virulence factors can enhance the pathogenic potential [20].

Mobile genetic elements such as plasmids, transposons, phages and pathogenicity islands play a key role in virulence [21]. Unlike studies from Europe and North America, there were only 4/136 O157:H7 serotypes, with O26:H11 (9/136), O115:H5 (9/136) and O63:H6 (8/136) being the predominant ones which rhyme existing epidemiological data [22, 23].

#### 3.2.2 AMR Profiles

**FIGURE 7:**
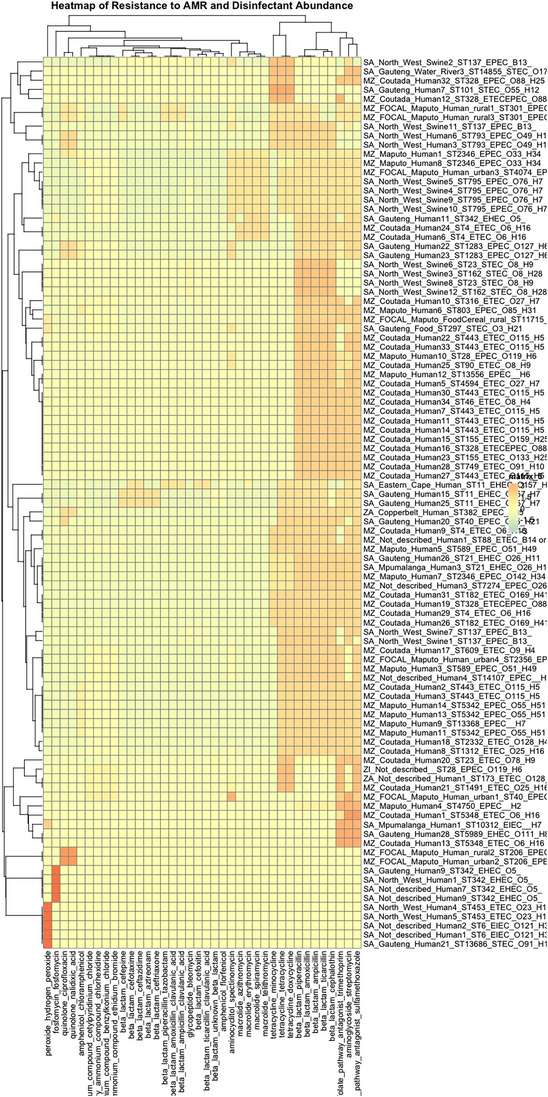
Heatmap of resistance to antimicrobial and disinfectants among DEC isolates from Southern Africa region. The names have five parts: ZA = Zambia, MZ = Mozambique, SA = South Africa and MZ_FOCAL = the Mozambique FOCAL project. The second part of the names = the provinces (regions) within these countries. The third part = the source of the samples. The fourth = the ST number. The fifth part = the pathotype. The sixth part = the O:H antigen.

A heatmap showing the likelihood and abundance of AMR and disinfectant‐associated genes among the 136 DEC isolates has been presented in Figure 7. Of these, 97 carried genes conferring resistance to at least one antimicrobial agent or disinfectant. The clustering revealed that resistance profiles were associated with both the type and abundance of the resistance genes. The proportion of DEC isolates resistance to specific antimicrobials or disinfectants is detailed in Figure S7. Notably, clustering appeared to be driven primarily by the geographical location, whereas the ST number, pathovar and O:H antigen played a lesser role.

The most frequently identified resistance genes were those associated with folate pathway antagonists (sulfamethoxazole—55.9% and trimethoprim (49.3), as well as β‐lactams (amoxilin—54.4%, ampicillin—54.4%, piperacillin—54.4%, tircarcillin—52.9 and cephalothin—52.2%). Additionally, resistance to aminoglycosides (streptomycin—55.1%) and tetracycline (doxycycline and tetracycline, both 38.2%) was prevalent, suggesting the presence of MDR.

Identifying clusters with distinct virulence and resistance profiles highlights the genetic diversity among DEC isolates and underscores the need for targeted surveillance and intervention strategies. The study found resistance to some antibiotics in the reserved category such as aztreonam, ceftazidime and fosfomycin, and some in the watch category such as azithromycin, cefotaxime and piperacillin + tazobactam according to the WHO AWaRe (Access, Watch, Reserve) guidelines (https://list.essentialmeds.org/antibiotics/watch).

The presence of MDR and ESBL‐producing DEC isolates in the Southern African region poses a public health concern as previously reported [24]. Similar to other studies, AMR to fourth‐generation cefepime and third‐generation ceftazidime, cefotaxime and ceftriaxome has been previously reported in the Southern Africa region [25, 26, 27].

**FIGURE 8:**
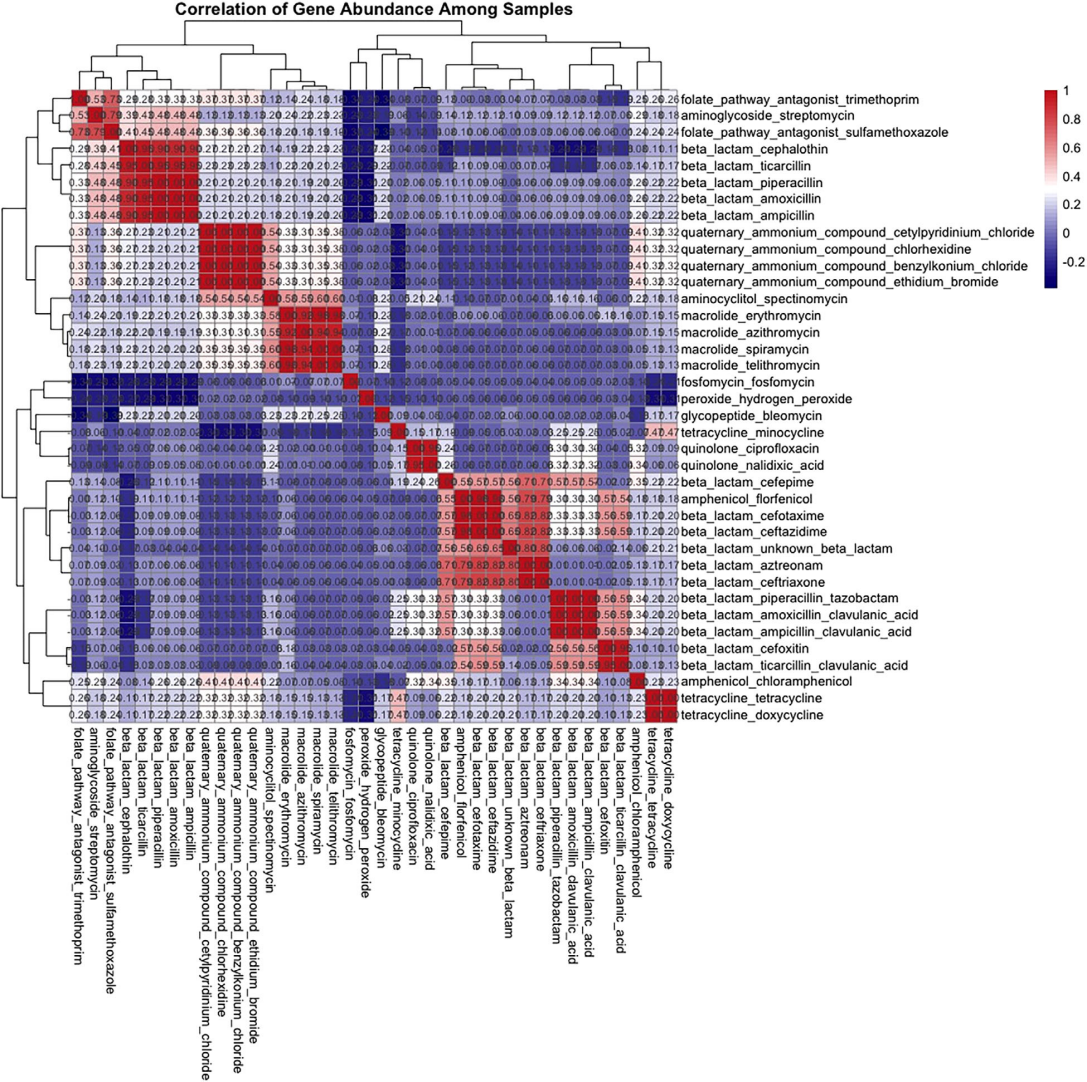
Correlation matrix of the abundance patterns of resistance to various antimicrobials and disinfectants among the DEC isolates.

Figure 8 presents the correlation matrix showing the abundance patterns of resistance to various antimicrobials and disinfectants among the DEC isolates. The correlation values ranged from −0.4 to 1, indicating relationships from weak negative to perfect positive correlations. Notably, resistance to disinfectants such as quaternary ammonium compounds and peroxides had a weak negative correlation to manly β‐lactams antimicrobials.

Only 12 of the 136 DEC isolates had chromosomal point mutation, as presented in Table 1. The identified mutations in the genes *gyrA*, *parC* and *parE* are well‐known markers for quinolone resistance (nalidixic acid and ciprofloxacin) which is consistent with the observed resistance profiles. In addition, all the isolates that conferred *gyrA*, *parC* and *parE* mutations were human isolates. Transferred genes are primarily concentrated in about 1% of chromosomal regions, known as hot spots, and their presence highlights extrachromosomal elements in integrating adaptive resistance genes, as suggested [28].

**TABLE 1:** Chromosomal point mutation conferring antimicrobial resistance of diarrhoeagenic *Escherichia coli* (DEC) isolates from the Southern Africa region.

	Sample	Gene	Mutation	Resistance	PubMed identifier
1	ZA_Copperbelt_Human	*gyrA*	gyrA p.S83L	Nalidixic acid, Ciprofloxacin	8891148
2	MZ_Maputo_Human1	*gyrA*	gyrA p.S83A	Nalidixic acid, Ciprofloxacin	12654733
3	ZA_Not_described_Human2	*parE*	parE p.I355T	Nalidixic acid, Ciprofloxacin	28598203
4	SA_Gauteng_Human22	*gyrA*	gyrA p.S83L	Nalidixic acid, Ciprofloxacin	8891148
5	SA_Gauteng_Human23	*gyrA*	gyrA p.S83L	Nalidixic acid, Ciprofloxacin	8891148
6	SA_North_West_Human6	*parC*	parC p.A56T	Nalidixic acid, Ciprofloxacin	12654733
7	MZ_FOCAL_Maputo_Human_rural2	*parC*	parC p.A56T	Nalidixic acid, Ciprofloxacin	12654733
8	MZ_FOCAL_Maputo_Human_urban2	*parC*	parC p.A56T	Nalidixic acid, Ciprofloxacin	12654733
9	MZ_FOCAL_Maputo_Human_rural1	*gyrA*	gyrA p.S83L	Nalidixic acid, Ciprofloxacin	8891148
		*parC*	parC p.S80I	Nalidixic acid, Ciprofloxacin	8851598
10	MZ_FOCAL_Maputo_Human_rural3	*gyrA*	gyrA p.S83L	Nalidixic acid, Ciprofloxacin	8891148
		*parC*	parC p.S80I	Nalidixic acid, Ciprofloxacin	8851598
11	MZ_Not_described_Human2	*parE*	parE p.I355T	Nalidixic acid, Ciprofloxacin	28598203
12	SA_North_West_Human3	*parC*	parC p.A56T	Nalidixic acid, Ciprofloxacin	12654733

*Note:* The names have three parts: ZA = Zambia, MZ = Mozambique, SA = South Africa and MZ_FOCAL = our current FOCAL project. The second part = the provinces (regions) within these countries. The third part = the sources.

**FIGURE 9:**
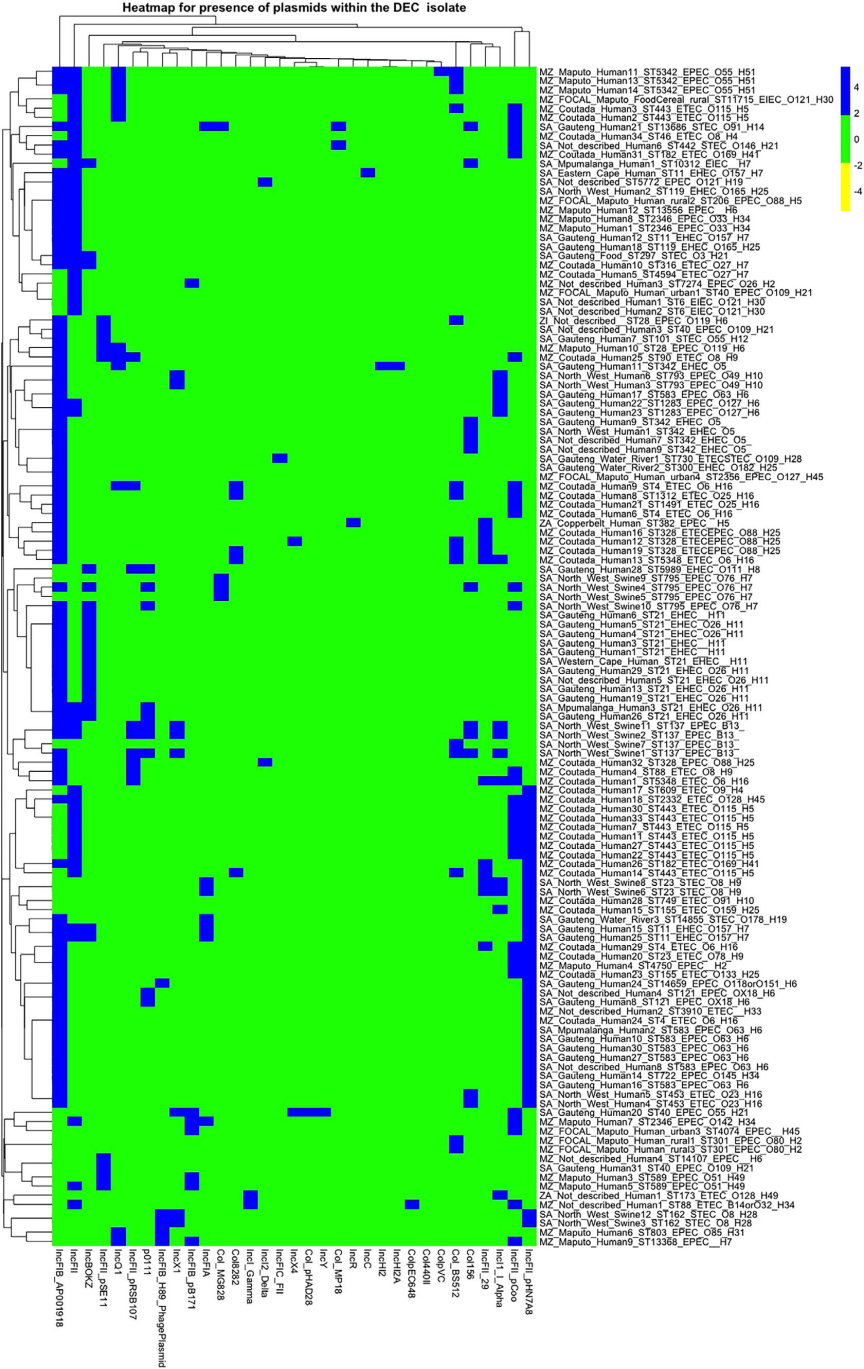
Hierarchical clustering of DEC isolates from the Southern Africa region based on the presence of plasmids. The names have five parts: ZA = Zambia, MZ = Mozambique, SA = South Africa and MZ_FOCAL = Mozambique FOCAL project. The second part of the names = the provinces (regions) within these countries. The third part = the source of the samples. The fourth = the ST number. The fifth part = the pathotype. The sixth part = the O:H antigen. DEC, diarrhoeagenic *Escherichia coli*.

3.2.2.1 Plasmids. A heatmap of plasmid harbouring AMR present in the DEC isolates from the Southern Africa region has been presented in Figure 9. The top 10 most abundant plasmid genes within the DEC isolates have been presented in Figure S8. Eight of the 136 DEC isolates had no plasmids, and of the eight, four were from the current research. IncFIB(AP001918) (23.06%), IncFII (12.78%), IncFII(pHN7A8) (9.27%), IncFII(pCoo) (8.77%) and IncB/O/K/Z (6.02%) were the most abundant plasmid. IncFIA, IncFIB, IncFIC and IncB/O have all been reported to be capable of carrying transfer, MDR and virulence‐associated genes and were all observed in this project [29]. The IncFII plasmids have been reported to be abundant among the resistance plasmids in Enterobacteriaceae from different sources, with their occurrence associated with AMR [30]. IncF plasmids have been reported to mobilize high‐pathogenicity islands [21]. IncFIB plasmids contribute to resistance to antibiotics such as β‐lactams, quinolones, aminoglycosides, tetracyclines and phenicols [31]. The distribution of plasmids among the DEC isolates was influenced by geographical location, suggesting horizontal plasmid transfer within regions.

**FIGURE 10:**
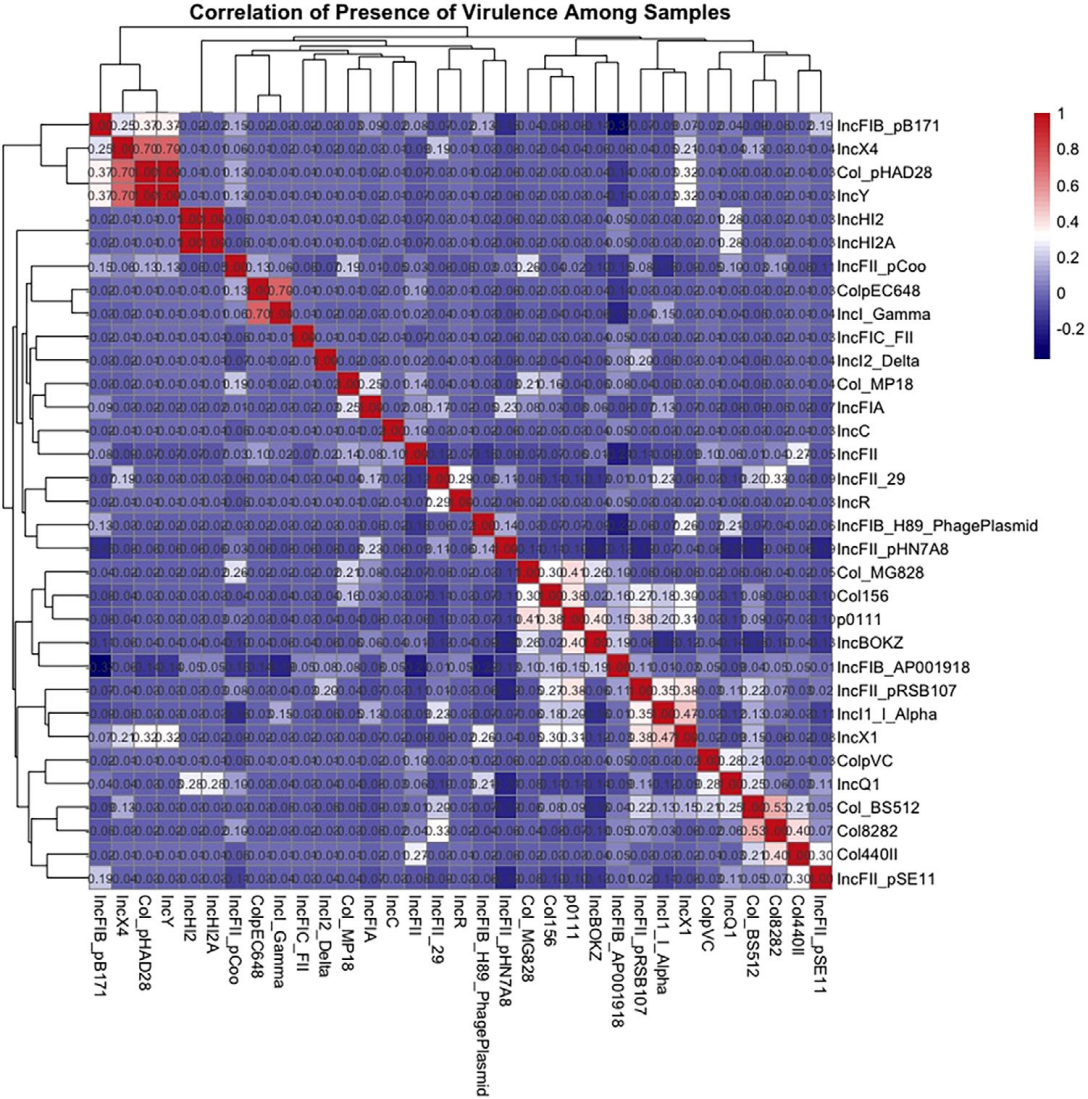
Correlation matrix showing the presence of plasmids among 136 DEC isolates from the Southern Africa region.

A correlation matrix of DEC isolates from the Southern Africa region based on the presence of plasmids has been presented in Figure 10. It was observed that Inc‐type plasmids generally exhibited a weak negative correlation with other Inc plasmids except for IncH12 and IncH12A. In addition, Inc‐type showed weak correlations with Col‐type and other non‐Inc plasmids. However, notably strong positive correlations were observed among IncX4, Col_pHAD28 and IncY plasmids, as well as ColpEC648 and IncI_Gamma plasmids, indicating they frequently co‐occur within the same isolates.

## 4 Conclusion

This study explored the genetic diversity, virulence‐associated determinants and AMR patterns of DEC isolates from Southern Africa using WGS data. By analysing isolates from foods consumed by children under five and faecal samples from those children in Maputo, alongside publicly available genomic data from the SADC region, this study reveals region‐specific genetic patterns, shared lineages and potential transmission pathways. The observed diversity in virulence‐associated factors and antimicrobial and disinfectant resistance highlights the capacity of DEC to adapt and persist across various ecological niches.

By applying unsupervised learning approaches, we identified epidemiologically relevant clusters based on geography, resistance patterns and transmission pathways. Clustering based on rST, MLST, cgMLST, wgMLST and O:H serotypes revealed the presence of closely related clades across different geographic locations, sources and collection periods, suggesting both localized contamination and broader regional dissemination. Evidence of horizontal gene transfer through shared virulence and resistance‐associated genes, chromosomal mutations and plasmids further underscores the dynamic evolution of DEC in response to environmental and anthropogenic pressures.

These findings provide critical genomic insights that can inform public health surveillance, outbreak detection and antimicrobial stewardship across Mozambique and the broader Southern African region. Integrating local and regional genomic data is essential to understanding DEC transmission dynamics and guiding effective interventions to reduce its impact on vulnerable populations, particularly young children.

## Author Contributions

**Josphat Gichure:** Conceptualization (equal); Formal analysis (equal); Methodology (equal); Writing original draft (equal); Writing review and editing (equal). **Tine Hald:** Conceptualization (equal); Writing review and editing (supporting); Funding acquisition. **Elna Buys:** Conceptualization (equal); Methodology (supporting); Supervision (lead); Writing review and editing (supporting).

## Ethics Statement

The DEC isolates collected from Mozambique are from the Foodborne Disease Epidemiology, Surveillance, and Control in African LMIC‐FOCAL project. Ethical clearance (registration number: CIBS FM & amp; HCM/092/2019) was obtained from the institutional health bioethics committee at the Faculty of Medicine/Maputo Central Hospital.

## Conflicts of Interest

The authors declare no conflicts of interest.

## Permission to Reproduce Material From Other Sources

We shall do a written request to EnteroBase. We acknowledge that these data were reproduced as per EnteroBase's data usage policies.

## Supporting information




**Supporting File 1**. puh270098‐sup‐0001‐SuppMat.docx

## Data Availability

The whole‐genome sequence (WGS) data generated in this study will be available in the National Center for Biotechnology Information (NCBI) Sequence Read Archive (SRA). The accession number will be shared, and data will be publicly accessible upon publication. Prior to publication, data can be made available upon reasonable request.
