# Supplementary material for: Exploring the Genetic Diversity, Virulence and Antimicrobial Resistance of Diarrhoeagenic Escherichia coli From Southern Africa Using Whole‐Genome Data
Source: Public Health Chall. 2025 Aug 12;4(3):e70098. doi: 10.1002/puh2.70098 (PMC12342054; doi:10.1002/puh2.70098)
Supplement: Supplementary file 1 — Supporting File 1. puh270098‐sup‐0001‐SuppMat.docx [file PUH2-4-e70098-s001.docx]

# Supplementary data

Table S1 Sample characteristics of 11 DEC isolate from Maputo, Mozambique

| Source | ID | Location | Description |
| --- | --- | --- | --- |
| Human | EC270 | Urban | 5 Months Female |
| Human | EC238 | Urban | 10 Months Female |
| Human | EC211 | Rural | 17 Months Female |
| Human | EC197 | Urban | 11 Months Female |
| Human | EC150 | Urban | 14 Months Male |
| Human | EC137 | Rural | 41 Months Female |
| Human | EC136 | Rural | 10 Months Female |
| Food | ET13 | Rural | Cereal |
| Food | EC16 | Rural | Drinking water |
| Food | EC5 | Urban | Combined_RiceFishStew |
| Food | EB1 | Urban | Drinking water |


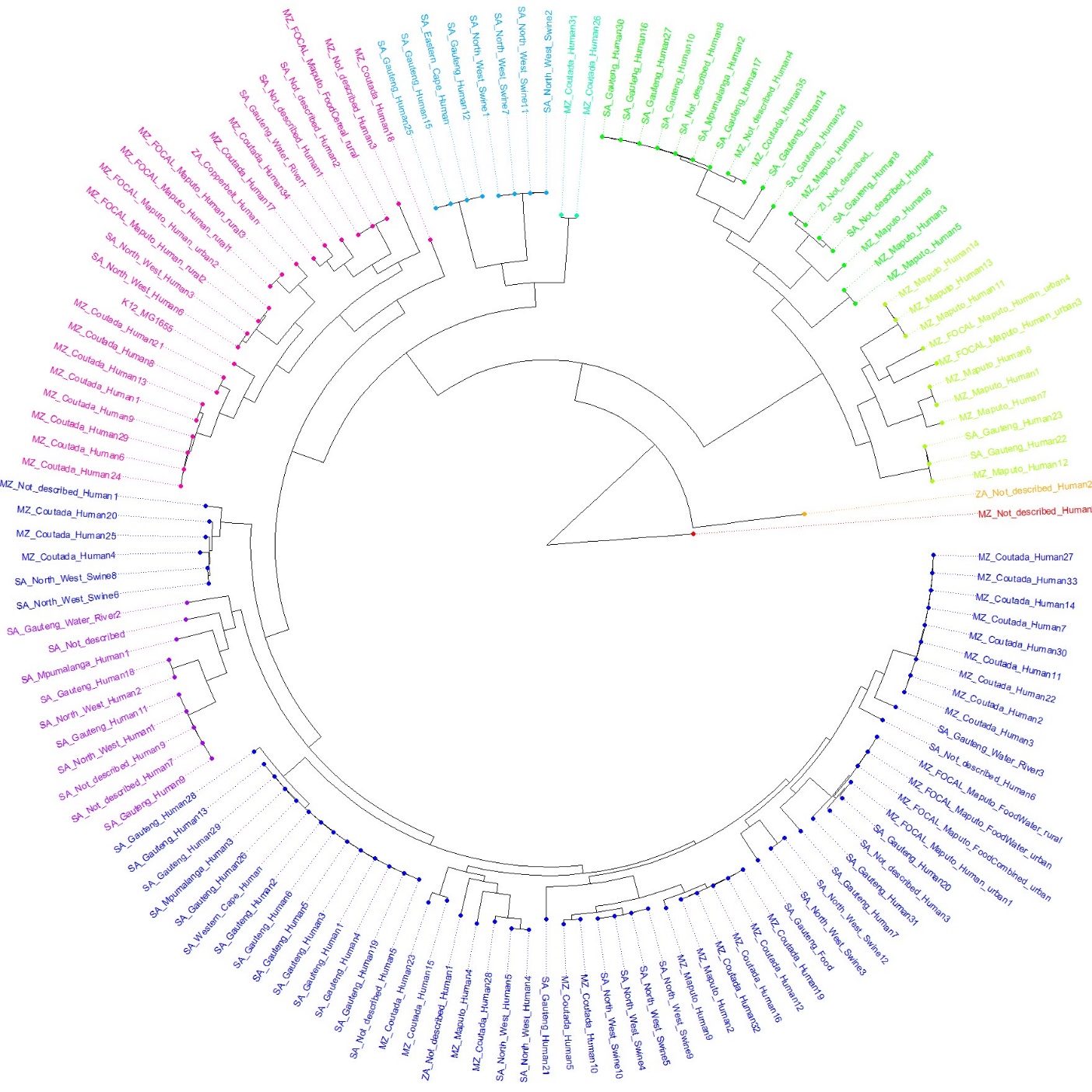


Figure S1 Phylogenetic relationships amongst 136 DEC isolates from the Southern Africa region were assessed using SNP. Hierarchical clustering (k-means) was performed using the cophenetic distance matrix. The tree was constructed using the UPGMA algorithm based on Euclidean. DEC isolates in the same cluster have similar colours. The names have three parts: ZA represents Zambia, MZ represent Mozambique, SA represents South Africa, and MZ_FOCAL represents the Mozambique FOCAL project. The second part of the names represents the provinces (regions) within these countries. The third part represents the source of the samples.


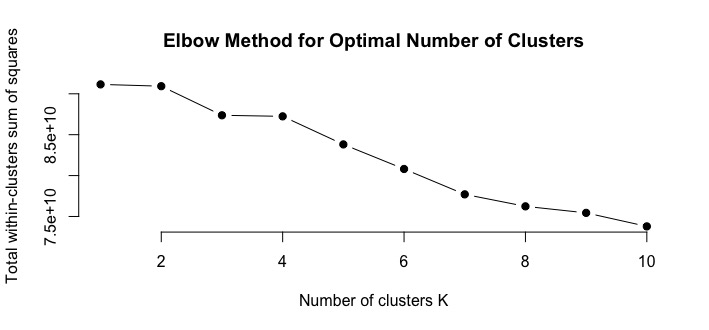


Figure S2 Number of optimal clusters for the 136 DEC isolates from Southern Africa region using the elbow method for Hierarchical clustering (k-means) performed using the cophenetic distance matrix.

Figure S3 Number of DEC isolates from the Southern Africa region in each cluster category using the elbow method for Hierarchical clustering (k-means) performed using the cophenetic distance matrix.


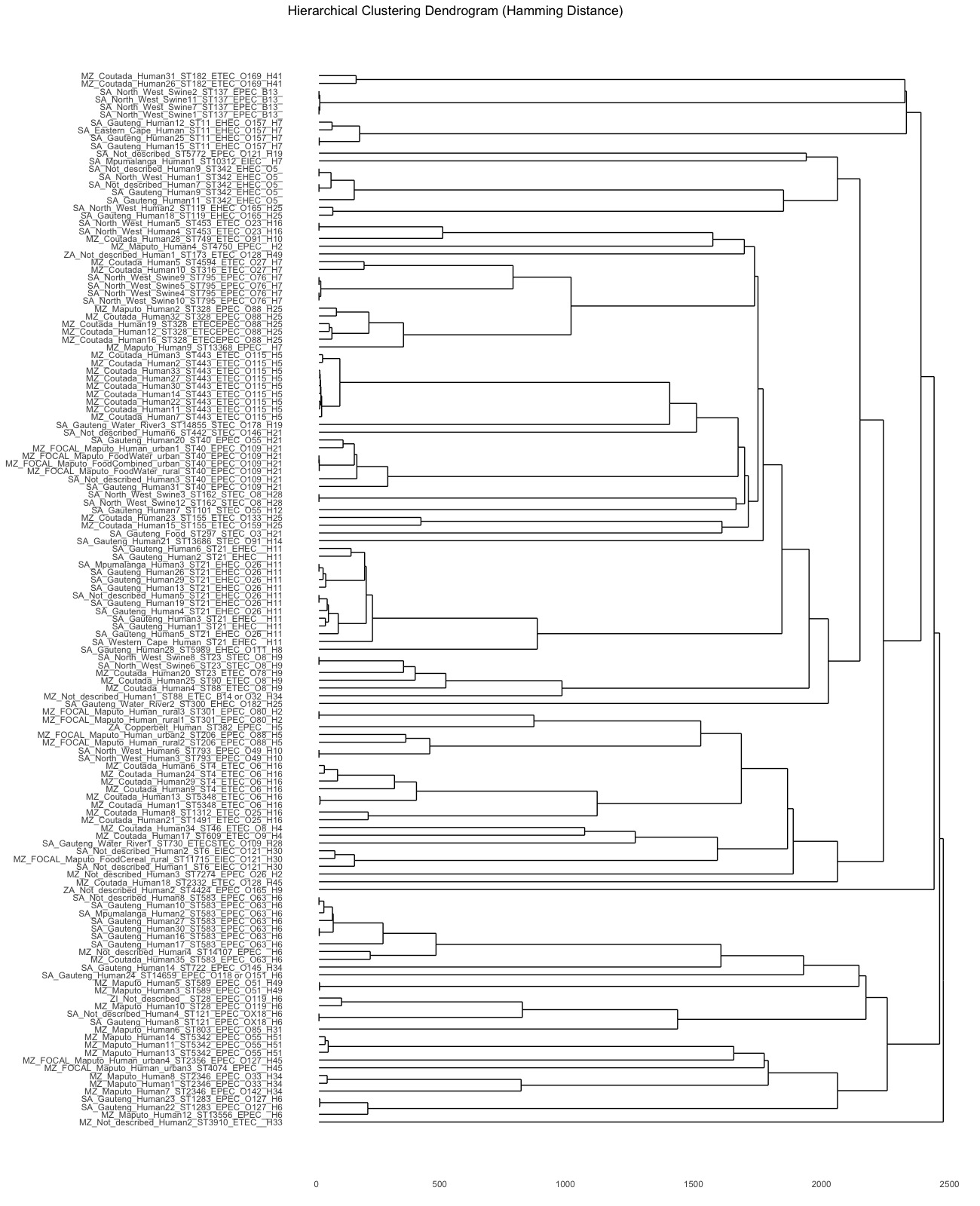
Figure S4 Clustering patterns based on the 2,513 core genomes within the 136 DEC isolates. The names have five parts: ZA represents Zambia, MZ represent Mozambique, SA represents South Africa, and MZ_FOCAL represents the Mozambique FOCAL project. The second part of the names represents the provinces (regions) within these countries. The third part represents the source of the samples. The fourth represents the ST number. The fifth part represents the pathotype. The sixth part represents the O: H antigen.


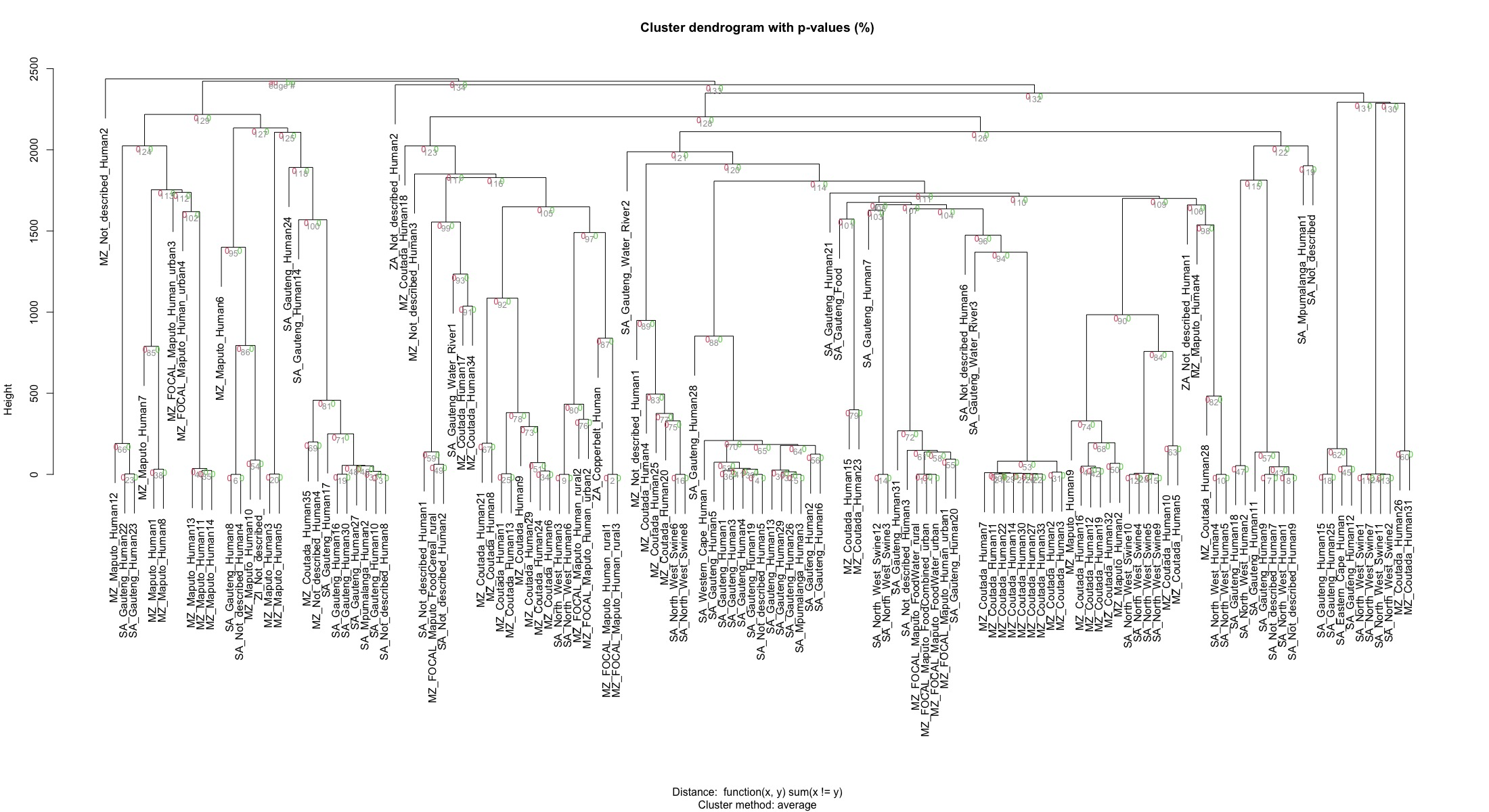


Figure S5 Cluster dendrogram with p-values of DEC isolates using cgMLST alleles from the Southern Africa region. The names have three parts: ZA represents Zambia, MZ represent Mozambique, SA represents South Africa, and MZ_FOCAL represents the Mozambique FOCAL project. The second part of the names represents the provinces (regions) within these countries. The third part represents the source of the samples.


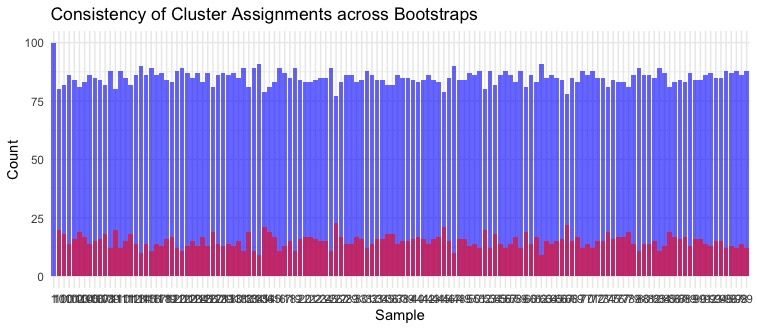


Figure S6 Consistency of cgMLST cluster assignment across bootstraps for the 136 DEC isolates from the Southern Africa region

Figure S7 The proportions of DEC isolates with antimicrobial or detergent resistance gene (n=136) from Southern Africa region

Figure S8 Top 10 most abundant plasmid genes within the 136 DEC isolates from the Southern Africa region
